# Assessing Antibiotic-Resistant Genes in University Dormitory Washing Machines

**DOI:** 10.3390/microorganisms12061112

**Published:** 2024-05-30

**Authors:** Wenbo Chen, Yu Zhang, Jiandui Mi

**Affiliations:** 1 State Key Laboratory for Animal Disease Control and Prevention, College of Veterinary Medicine, Lanzhou University, No. 222 South Tianshui Road, Lanzhou 730000, China; wenbo.chen.22@alumni.ucl.ac.uk; 2 Division of Bioscience, University College London, London WC1E 6BT, UK; 3 Guangdong Provincial Research Center for Environment Pollution Control and Remediation Materials, College of Life Science and Technology, Jinan University, Guangzhou 510632, China; zhangyu971019@foxmail.com; 4 Gansu Province Research Center for Basic Disciplines of Pathogen Biology, Lanzhou 730046, China

**Keywords:** washing machine, antibiotic resistance gene, bacterial community, mobile genetic element

## Abstract

University dormitories represent densely populated environments, and washing machines are potential sites for the spread of bacteria and microbes. However, the extent of antibiotic resistance gene (ARG) variation in washing machines within university dormitories and their potential health risks are largely unknown. To disclose the occurrence of ARGs and antibiotic-resistant bacteria from university dormitories, we collected samples from washing machines in 10 dormitories and used metagenomic sequencing technology to determine microbial and ARG abundance. Our results showed abundant microbial diversity, with Proteobacteria being the dominant microorganism that harbors many ARGs. The majority of the existing ARGs were associated with antibiotic target alteration and efflux, conferring multidrug resistance. We identified tnpA and IS91 as the most abundant mobile genetic elements (MGEs) in washing machines and found that *Micavibrio aeruginosavorus*, *Aquincola tertiaricarbonis*, and *Mycolicibacterium iranicum* had high levels of ARGs. Our study highlights the potential transmission of pathogens from washing machines to humans and the surrounding environment. Pollution in washing machines poses a severe threat to public health and demands attention. Therefore, it is crucial to explore effective methods for reducing the reproduction of multidrug resistance.

## 1. Introduction

The washing machine is a commonly used household appliance in daily life. However, numerous studies have indicated the widespread presence of harmful microorganisms in washing machines [1,2]. Complete water drainage after the washing cycle can be challenging, and the machines are often situated in damp environments, creating ideal conditions for microbial growth [3]. Microbial contamination in washing machines can come from various sources, including human skin, mucosal biota, bodily excretions, and various microorganisms from the environment [4]. After laundering, some microbes inevitably remain in the washing machine and gradually multiply. Surviving bacteria can form biofilms, which serve as reservoirs for pathogenic microorganisms and have a higher resistance to detergents that have been used [5,6]. This can result in the secondary contamination of washed clothes with pathogenic bacteria. One study has described how washing machines can lead to cross-contamination with a single strain of a multidrug-resistant bacterium (*Klebsiella oxytoca* sequence type 201) in the healthcare sector [7]. Direct contact with contaminated clothes can lead to cross-contamination between families, posing a potential risk to human health.

The environment houses a wide variety of microorganisms, many of which harbor antibiotic-resistance genes (ARGs). These genes can originate from both internal resistance and external sources of microorganisms in the environment. It is increasingly recognized that ARGs are not only present in clinical settings, but also in animals, plants, and the environment. Clinical pathogens, as well as pathogenic, commensal, and environmental bacteria, mobile genetic elements (MGEs), and bacteriophages, form a reservoir of ARGs known as the resistome. Pathogenic bacteria can acquire resistance through horizontal gene transfer (HGT) from this resistome [8,9]. The transmission of ARGs from non-pathogenic bacteria to pathogens can cause more ecotoxicological effects than antibiotics themselves in the natural environment [10]. In a study, samples were collected from shower drains, dishwashers, washing machines, and the domestic environment, revealing the domestic environment as a habitat for β-lactamase genes and β-lactam-resistant bacteria [11]. Additionally, it has been demonstrated that antibiotic-resistant bacteria and β-lactamase genes can persist in domestic washing machines [12]. Therefore, it is reasonable to assume that microorganisms carrying a broad range of ARGs could be present in washing machines, and they can promote the transfer of plasmid-mediated resistance genes.

University dormitories represent densely populated environments where students live, study, and socialize, and dormitory washing machines are potential sites for the spread of bacteria and microbes. Given that a large number of students using the same washing machine may release various microorganisms and bacteria during the washing process, urgent attention is warranted to research ARG pollution within washing machines in university dormitories. Metagenomics is a new approach to comprehensively sample all genes present [13]. While microbiome studies on ARGs by metagenomic sequencing have extensively investigated environments like wastewater [14,15], soil [16], manures [16], sewage [17], and other environments, there has been a relatively notable scarcity of studies examining the microbiome and ARGs within households, particularly in the context of washing machines [18,19]. Specifically, the broader spectrum of ARGs and their implications for antimicrobial resistance dissemination in communal living spaces remain unexplored. Therefore, our study aims to investigate the occurrence of ARGs and antibiotic-resistant bacteria in dormitory washing machines by metagenomic sequencing technology. This study’s findings will be significant for public health by informing strategies to curb antibiotic resistance in communal settings like university dormitories, thereby mitigating the spread of resistant bacteria beyond human populations.

## 2. Material and Methods

### 2.1. Sample Collection

Six male and six female dormitories located at South China Agricultural University (Guangzhou, China) were randomly selected for this study. Each dormitory housed six individuals. All washing machines used in this study were fully automatic top-loading washing machines without drying functionality, commonly found in many households and dormitories. The maximum capacity of these washing machines ranged from 5 to 8 kg. The washing machines in the dormitories were exclusively used by residents. Each machine had an average frequency of use of once a day, and they were in operation for a duration of 2–4 years. We conducted our sampling in May 2021 during the school’s epidemic control measures, during which individuals’ activities were mainly confined to Guangzhou City for an extended period. Due to this sampling timeframe, people tended to wear summer clothing such as dresses, skirts, T-shirts, and shorts, primarily made of cotton and polyester.

Residues on the washing machine filters were collected several times using sterile cotton swabs to ensure that the samples weighed approximately 1.0 g. The collected samples, consisting of residue accumulated on the filter screen due to prolonged drum washing machine use, were stored in sterile tubes and promptly transferred to a −80 °C refrigerator for subsequent DNA extraction.

### 2.2. DNA Extraction

The DNA in the filter screen samples of the washing machine was extracted using the QIAamp Power Fecal Pro DNA Kit from Qiagen (Hilden, Germany). The concentration and quality of the DNA were assessed using a Qubit 3.0 from Thermo Fisher Scientific (USA), and its integrity was examined by Fragment Analyzer 5400 (Agilent Technologies, Santa Clara, CA, USA). The results showed that most of each DNA fragment from female and male dormitories was below 500 bp. Therefore, two samples were eliminated prior to the construction of metagenomic sequencing libraries. The DNA samples were then stored at −20 °C for further analysis. The sequencing libraries were prepared using the NEBNext^®^ Ultra™ DNA Library Prep Kit (E7645S, NEB, Ipswich, MA, USA), and all sequencing was performed using the NovaSeq 6000 system from Novogene Co., Ltd. (Beijing, China).

### 2.3. Metagenomic Assembly, ARG and MGE Analysis

After eliminating unqualified samples for classification, we analyzed 10 filter screen samples from the washing machine. The raw sequence was used to quality control with Fastq v0.19.7 and the following paired reads were discarded to generate clean reads: (1) If either one read contains adapter contamination; (2) If more than 10% of bases are uncertain in either one read; (3) If the proportion low quality (Phred quality < 5) bases is over 50% in either one read. We generated a total of 223,993,242 clean reads, with an average of 22,399,324 reads per sample (range from 20,233,272 to 25,442,726) (Table S1). The kraken2 (v2.1.2) [20] and bracken (v2.9) [21] with default parameters were used to calculate the relative abundances of the microbiome at different taxonomy levels (such as phylum and species level) within entire metagenomic reads of samples according to procedures as previous report [22]. The alpha diversity of the microbiome in different samples was calculated using KrakenTools [22]. We assembled the clean data with Megahit (version 1.2.9) [23] in the paired-end mode using kmer lists 21, 29, 39, 59, 79, 99, 119, and 141. We predicted genes using the Prodigal “pmeta” model (version 2.6.3) [24] and used CD-HIT (version 4.8.1) to cluster the nonredundant gene catalog with default parameters [25]. We counted unique genes using Salmon (version 1.1.0) with “meta” and “mimicBT2” models [26] to calculate relative gene abundance. We identified predicted unique genes as ARG or MGE-like genes by searching proteins against the Comprehensive Antimicrobial Resistance Database (CARD) [27] and MGE database [28] using BLASTP with an e-value ≤ 10^−5^, coverage ≥ 60%, and identity ≥ 70%.

To remove technical biases arising from differences in the depth of sequencing and gene length, we used transcripts per million (TPM) to calculate gene counts generated by Salmon, based on the formula by Zhang et al. (2022). Open reading frames (ORFs) which were identified on ARG-carrying contigs were classified against the NCBI non-redundant (NR) protein database using diamond with blastp with e-value ≤ 10^−5^, with an identity and query coverage threshold of 70% and 60%, respectively. Finally, the count number of ARGs belonging to different species was calculated by summing them together for the same species. We assembled contigs into metagenome-assembled genomes (MAGs) of each sample using MetaWRAP (version 1.3.1) with the “metabat2” method [29] and dereplicated the MAGs using dRep (version 3.2.2) with default parameters [30]. We obtained a total of 201 MAGs with an estimated completeness of ≥50% and contamination of ≤5%, including 97 MAGs with an estimated completeness of ≥90%. We performed taxonomic assignment of the MAGs using GTDB-Tk (version 1.5.0) with default parameters [31]. We identified ARGs and MGEs carried by MAGs using BLASTX against the Comprehensive Antimicrobial Resistance Database (CARD) [27] and MGE database [28] with an e-value cutoff of 10^−5^, 70% similarity, and 60% query coverage. The count number of ARGs and MGEs carried by MAGs was calculated by summing together according to the categories of ARGs, MGEs and bacterial taxonomy at the genus and species level.

### 2.4. Statistical Analysis and Result Visualization

Analysis of Similarities (ANOSIM) was performed to assess group differences. The significant difference between genders was calculated using a Student’s *t*-test. A *p*-value < 0.05 was considered to be statistically significant. The non-metric multidimensional scaling (NMDS) was calculated and plotted using R (v4.2.2). The circle richness and abundance figures were also created using R (v4.2.2). The figures of the bar, box, and heatmap were plotted with GraphPad Prism (v9.0.0).

## 3. Results and Discussion

### 3.1. Diversity and Abundance of ARGs

In this study, a total of 366 classified ARGs were detected. Gender was taken into consideration to assess the effect on the diversity and abundance of ARGs. As shown in Figure S1A, the stress was less than 0.02, indicating the reliability of the results. The NMDS plot of gene profiles (β-diversity) showed that the OTU intervals of washing machine contents for both male and female groups intersected, indicating no significant difference in the abundance of detected ARGs between genders (Figure S1A). In terms of α-diversity analysis, the Chao1 and Shannon index showed that the diversity of ARGs between the two groups was not significantly distinct (Figure S1B). Furthermore, the top three most abundant types of ARGs were elfamycin, multi-drugs, and aminoglycoside, with tetracycline, glycopeptide, peptide, aminocoumarin, fusidic acid, fluoroquinolone, isoniazid, and others also detected in this study (Figure S1C). Characterizing the function of the ARGs (Figure S1D), antibiotic target alteration and antibiotic efflux were identified as the primary microbial resistance mechanisms, followed by multi-mechanisms, antibiotic target protection, antibiotic inactivation, antibiotic target replacement, and reduced permeability to the antibiotic. Furthermore, the abundance of ARGs in the washing machine samples from the male group was found to be higher compared to the female group. Gender differences in domestic ARGs are not typically a focus of research in the field. After checking other domestic ARG studies, it was found that toothbrushes belonging to individuals identifying as women harbored more diverse microbial communities compared to those owned by men. However, gender identity did not show a significant correlation with the alpha diversity of taxonomic or ARG profiles in oral metagenomes [32].

**Figure 1:**
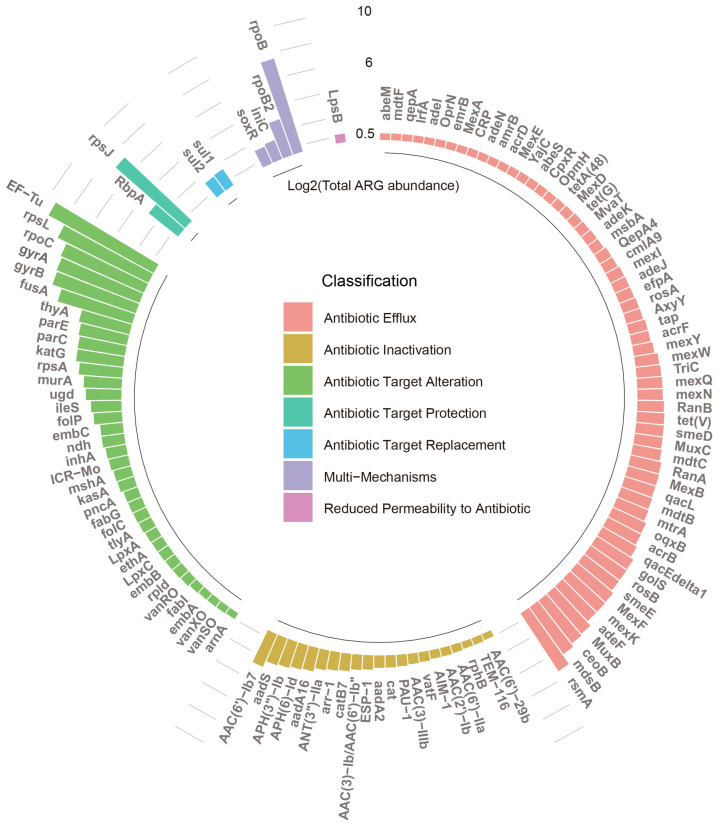
Log2-transformed number of total ARGs abundance associated with ARGs related to the microbial resistance mechanisms.

In this study, efflux contained the most diverse types of resistance genes (Figure 1), and it has also been found in other types of domestic samples [33]. Efflux pumps in bacteria efficiently expel a variety of antibiotics from the cell, preventing antibiotics from entering the cell or reducing their concentration within the bacterial cell. This system plays a key role in the resistance of many multi-resistant bacteria to multiple antibiotics or heavy metals. The change of target prevents antibiotics from binding to bacteria and inhibiting essential cellular components of bacteria, while not affecting the normal metabolic function of the target. Further analysis of the mechanisms and ARG subtypes revealed that the total ARG abundance was classified into seven microbial resistance mechanisms. Antibiotic target alteration in washing machines mainly includes EF-Tu, rpsL, rpoC, gyrA, gyrB, fusA, and others. The most abundant ARG EF-Tu is a prokaryotic protein synthesis factor, and its transition to an ARG highlights the adaptability of bacteria in the face of selective pressures, such as exposure to antibiotics [34]. The gyrA and gyrB genes, as mentioned earlier, are part of the DNA gyrase enzyme, and their abundance may be indicative of the genetic elements that confer resistance to antibiotics targeting DNA gyrase [35]. The genes rpsL and fusA, for translation, these deleterious effects are often associated with a reduction in the bacterial growth rate due to reduced protein production rates. These genes, which are associated with antibiotic target alteration, are of great importance due to their role in mediating resistance to antibiotics [36]. Higher abundances of rsmA and mdsB were observed for antibiotic efflux. Additionally, multi-mechanisms included the subtype rpoB, while antibiotic target protection included the subtype rpsJ, both of which had a high number of genes (Figure 1). The rpoB gene encodes the RNA polymerase β subunit, the primary target of rifampicin, a crucial drug in treating tuberculosis and other mycobacterial infections. This gene is universal across all bacteria. Mutations within the rpoB gene lead to changes in the protein’s structure, resulting in drug resistance [37].

### 3.2. Diversity and Abundance of MGEs

**Figure 2:**
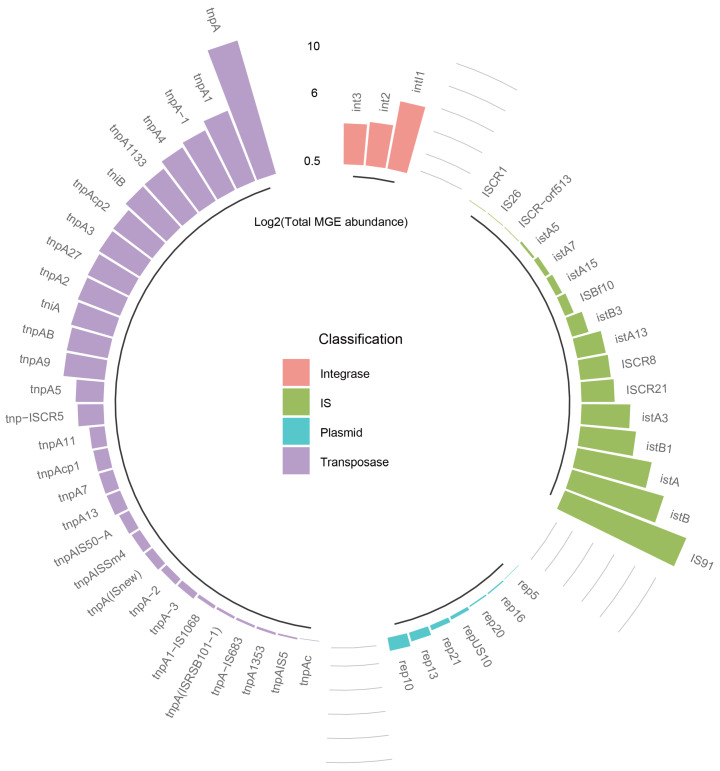
Log2-transformed number of total MGE abundance according to four different classifications. IS represents insertion sequence.

Given that the dissemination of ARGs is primarily propelled by MGEs, we then focused on their diversity and abundance. Figure S2 shows a clear difference in the MGE profile between the male and female groups, as evidenced by the significantly distinct NMDS plot based on Bray–Curtis distance (Figure S2A). However, microbial α-biodiversity analysis using Chao 1 and Shannon index revealed that the diversity of MGEs did not differ significantly between the two groups (*p* > 0.05) (Figure S2B). In total, a variety of MGE species were detected in the samples. Further examination of MGE abundance showed that transposase genes, including tnpA, tnpA1, tnpA-1, and tnpA4, as well as insertion sequence (IS) genes, particularly IS91, istB, istA, and istB1, comprised a large proportion of the total MGE abundance (Figure S2C and Figure 2). Additionally, the integrase gene intl1 was more abundant than the integrase genes, as indicated in Figure 2. The main mechanism for acquiring antibiotic resistance is through the horizontal gene transfer of ARGs, which is mediated by MGEs. The high abundance and diversity of bacteria in washing machines create an environment conducive to the horizontal transfer of ARGs. Transposon tnpA is the most frequently detected MGE and has been found in various environmental samples [38,39]. Other MGEs with high abundance include IS91, IST A, and IST B, which are classified as IS and can transfer DNA between different locations on the same or different chromosomes. These MGEs are commonly found in wastewater treatment plants [40]. Some of the indexes detected in this study are similar to those found in wastewater samples. It is evident that MGEs played a crucial role in the dissemination of ARGs in previous studies, which qualitatively assessed the health risks associated with ARGs. MGEs, including transposons, plasmids, and integrons, significantly facilitate the horizontal transfer of ARGs between bacteria. With their transposition and replication mechanisms, MGEs act as vehicles for the swift dissemination of ARGs, thereby contributing to the adaptability and proliferation of antibiotic resistance within microbial communities [41].

### 3.3. Characterization of Bacterial Community

**Figure 3:**
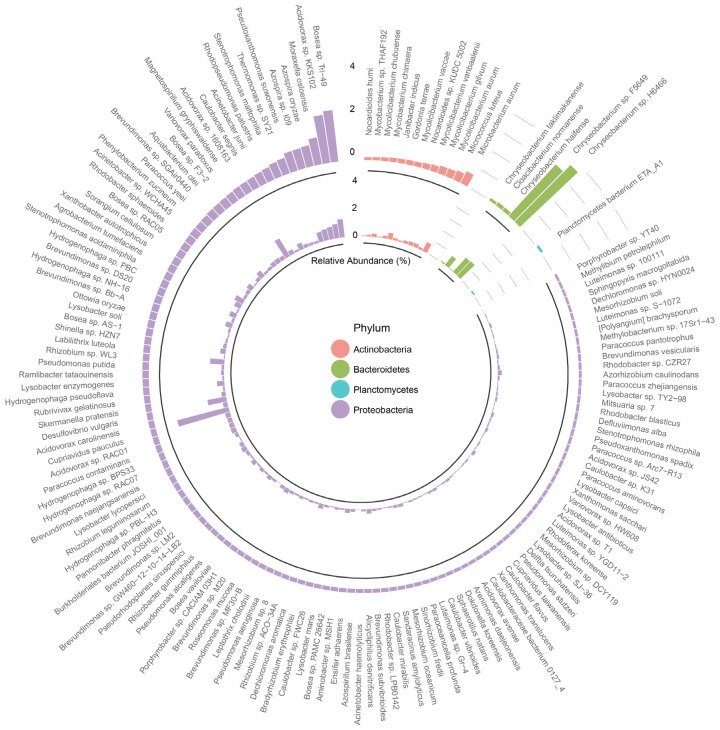
Relative bacterial abundance at the species level for male (outer ring) and female (inner ring) samples, respectively.

After annotating the samples from 10 washing machines based on their effect sequences, the composition and abundance of bacteria at phylum and species level in the washing machine were conducted as shown in Figure 3 and Figure S3. Next, we analyzed the annotation results and identified the species with the highest abundance in each group at the phylum classification level and drew a relative abundance histogram of the species. The bacterial species in the female group were widely distributed and showed great diversity, while the male group had relatively small differences within the group. As evident from the statistical test using the ANOSIM method (*p* = 0.675), the differences in bacterial species between the two groups were not found, so the species were concentrated as a whole in the NMDS plot based on Bray–Cutis distance (Figure S3A). Moreover, gender had no significant impact on the bacterial community across different sampling types as shown in Figure S3B (*p* > 0.05).

Based on the phylum-level analysis of all samples, the following bacterial phyla were identified: Proteobacteria, Actinobacteria, Bacteroidetes, Planctomycetes, and Firmicutes, along with other less frequently detected communities. Proteobacteria was the dominant phylum in both the female group and male group, accounting for 74.73% and 74.73% of the total sequences, respectively. Actinobacteria and Bacteroidetes were also abundant in the female washing machines, accounting for 16.76% and 5.17%, respectively. Actinobacteria and Bacteroidetes were also abundant in the male washing machines, accounting for 10.88% and 11.56%, respectively. The proportion of Actinobacteria was more than twice as abundant as Bacteroidetes in the female group, while in the male group, the proportion of Actinobacteria was slightly lower than that of Bacteroidetes (Figure S3C). The distribution of resistance genes is closely linked to changes in microbial communities, which makes it crucial to investigate the microbial community structures in different environments. The results showed that the most abundant microbial taxa were Proteobacteria, Actinobacteria, and Bacteroidetes. Proteobacteria and Actinobacteria are known to be important carriers of ARGs and are also detected as the most abundant bacterial phyla in many other environments, such as water supply pipes and networks [42,43,44,45]. Proteobacteria is associated with a range of diseases, including metabolic disorders, inflammatory bowel disease, and lung disease [46]. Actinobacteria can infect the oral, gastrointestinal, and genital mucous membranes of healthy individuals and are generally non-infectious [47]. However, actinomycetes-associated diseases can occur in certain hosts, such as those with lung parenchymal damage, chronic debilitating diseases, or who smoke and abuse alcohol, so it is important to be aware of their potential danger [48].

At the species level, the abundance of *Chryseobacterium* sp. *H6466*, *Chryseobacterium* sp. *F5649*, *Bosea* sp. *Tri−49*, and *Acidovorax* sp. *KKS102* were all extremely high in both gender groups, each accounting for more than 3%. However, there were also some notable differences in detail. For example, the proportion of *Cupriavidus pauculus* and *Variovorax paradoxus* was higher in the female group than in the male group (Figure 3). *Chryseobacterium* species, including *Chryseobacterium taklimakanense*, *Chryseobacterium haifense*, *Chryseobacterium* sp. *F5649*, and *Chryseobacterium* sp. *H6466*, are common opportunistic pathogens in clinical practice, capable of causing infections such as meningitis, cellulitis, and sepsis. It has also been reported that ARG-carrying MGEs of *Chryseobacterium* species may increase the probability of horizontal gene transfer, thereby promoting the propagation of new multidrug-resistant bacteria [49]. M. Boxberger isolated a *Chryseobacterium* genus strain from healthy human skin, which could be a potential source of these bacteria [50]. Due to their rapid growth and efficient membrane transport systems under in vitro conditions, *Chryseobacterium* species pose a threat to humans. Specifically, strains *F5649* and *H6466* have been reported as *Chryseobacterium hominis*, which can grow at temperatures of 30 and 37 °C (with optimal growth at 30 °C) [51]. This is named as such because most of the known isolates at the time of description were of human origin [52]. This could explain their high abundance in Figure 3. Additionally, *Cupriavidus pauculus*, which showed the largest difference between genders [53,54], is a widely distributed pathogen that can cause diseases such as ulcerative colitis [55], pneumonia [54,56], and histiocytic necrotizing lymphadenitis [57], especially in immunocompromised patients such as infants and the elderly.

### 3.4. The Abundance and Occurrence of Microbial Communities Associated with the ARGs and MGEs

**Figure 4:**
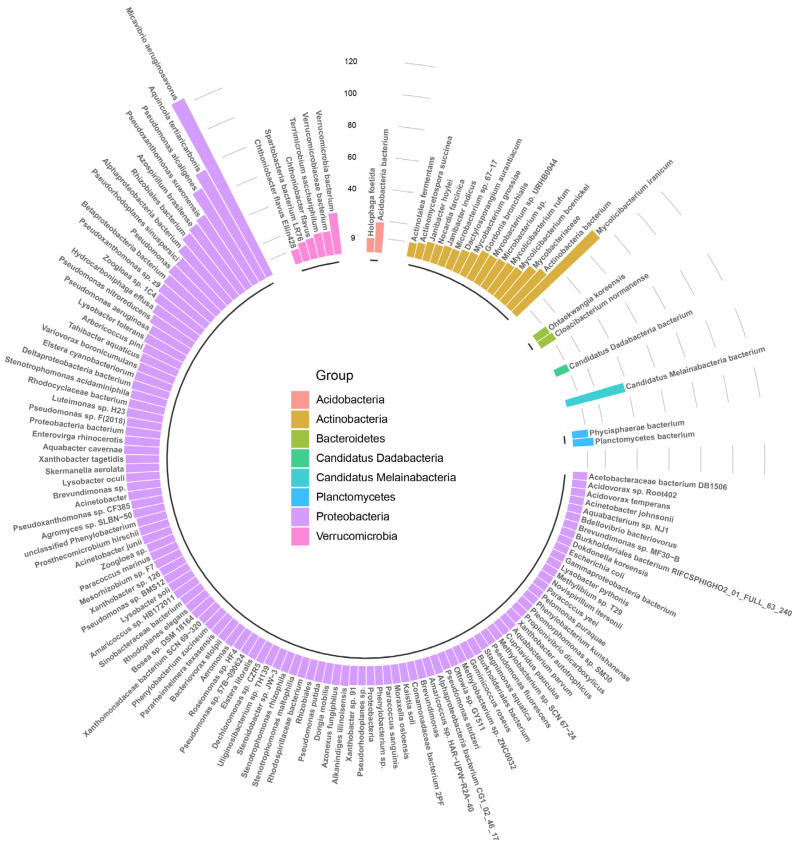
Number of antibiotic resistance genes (ARGs) identified from predicted open reading frames (ORFs) of contigs associated with primary bacterial species combined with the female and male groups.

We further examined the diversity of unique ARGs associated with each bacterium shown in Figure 4. As expected, Proteobacteria had the highest percentage of ARGs. Notably, Micavibrio aeruginosavorus had more than 120 distinct types of ARGs, followed by *Aquincola tertiaricarbonis*, *Pseudoxanthomonas suwonensis*, *Pseudomonas alcaligenes*, *Rhizobiales bacterium*, *Azospirillum brasilense*, and *Alphaproteobacteria bacterium*. Among *Actinobacteria*, *Mycolicibacterium iranicum* was found to harbor numerous types of ARGs.

*Micavibrio aeruginosavorus* is a Gram-negative predatory bacterium that selectively preys on other bacteria, including multidrug-resistant ones, by attaching to their surface and feeding on their cellular contents. This makes it a promising candidate for use as “live antibiotics,” which could reduce the reliance on traditional antibiotics and mitigate the problem of bacterial resistance [58,59]. Importantly, *Micavibrio aeruginosavorus* is non-pathogenic to humans. Interestingly, some of the bacteria that *Micavibrio aeruginosavorus* preys upon, such as *Stenotrophomonas maltophilia*, *Pseudomonas aeruginosa*, and *Serratia marcescens* [60,61], were also found in significant numbers in the washing machine samples shown in Figure 3. This may explain why *Micavibrio aeruginosavorus* has the most resistance genes among the bacteria analyzed. Moreover, *Micavibrio aeruginosavorus* has been noted to be “completely devoid of mobile genetic elements” and lacking in repetitive regions and pseudogenes, suggesting that the ARGs found in this bacterium may have originated from itself rather than being acquired from other bacteria [59,62].

In 2013, Shojaei first reported *Mycolicibacterium iranicum* [63], which is characterized by rapid growth and orange-pigmented scotochromogenic colonies. Studies have shown that it is associated with lung disease and peritonitis [63,64]. *Mycolicibacterium iranicum* is a non-tuberculous mycobacteria (NTM) [65], which refers to other mycobacteria in the *Mycobacterium* genus except *Mycobacterium tuberculosis* complex and *Mycobacterium leprae*. NTM is widely present in natural environments such as water, soil, and dust, and people can become infected with NTM from the environment, leading to disease. *Aquincola tertiaricarbonis* was first isolated from methyl tert-butyl ether (MTBE) contaminated groundwater in Germany and a wastewater plant in France [66]. MTBE, ethyl tert-butyl ether (ETBE), and amyl tert-butyl ether (TAME) are commonly found in groundwater but are difficult to degrade due to their ether bonds and tertiary carbon atoms. Muller [67] discovered that *Aquincola tertiaricarbonis* has a significant capacity to degrade the primary intermediate metabolites of these compounds, making it a promising candidate for bioremediation in similarly contaminated environments. *Pseudoxanthomonas suwonensis* is a bacterium that can degrade cellulose and produce dioxygenase to promote the biodegradation of many types of organic compounds. It is often found in solid waste and can enhance the rapid production of enriched biocompost [68]. According to one study, strains of this bacterium can be isolated from cotton waste composts [53]. Therefore, it is reasonable to suggest that *Pseudoxanthomonas suwonensis* may live in samples from washing machines.

**Figure 5:**
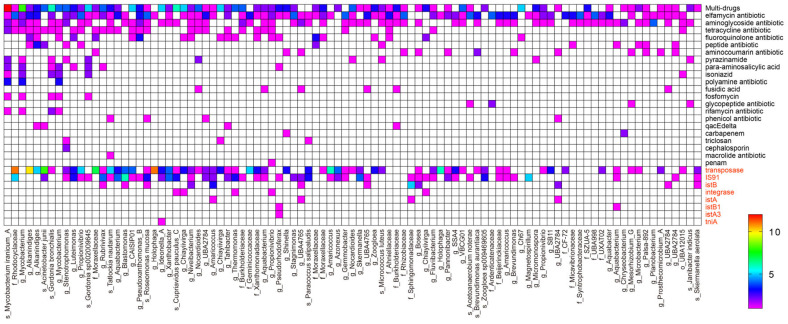
Heatmap displaying antibiotic resistance genes (ARGs) and mobile genetic elements (MGEs) associated with metagenome-assembled genomes (MAGs) above the quality with completeness of ≥50% and contamination of ≤5%. The names of genes marked in red and black represent MGEs and ARGs, respectively, on the ordinate.

Upon examining the species information, we can gain insight into which species harbor a higher abundance of resistance genes. As illustrated in Figure 5, multi-drug resistance was observed to be strongly correlated with *Mycobacterium*, particularly *Mycolicibacterium iranicum*. To further investigate the association between ARGs/MGEs and dominant bacterial taxa, we constructed co-occurrence patterns. By doing so, we hypothesized that co-occurrence patterns between ARGs and bacteria can provide valuable information regarding the potential host of ARGs, particularly in cases where there exists a strong and statistically significant positive correlation between ARGs and coexisting bacterial taxa. Our findings showed that transposase was closely linked to f_*Rhodocyclaceae*, g_*Alkanindiges*, s_*Acinetobacter junii*, and f_*Moraxellaceae*, while IS91 was correlated with f_*Rhodocyclaceae*, g_*Blastomonas* and g_*Magnetospirillum*, indicating that these bacteria are likely to promote the transmission of ARGs. Furthermore, several ARGs displayed significant positive correlations with individual genera, suggesting the presence of distinct ARGs within a single host bacterium.

Upon comparing the similarities in community diversity and ARGs between our study and previous research, we can infer that the ARGs identified in this study may have originated from bacteria associated with clothes and humans or could have resulted from water contamination or transmission by other environmental factors. Moreover, we observed that the dominant bacterial species and relative abundance of ARGs and MGEs remained unchanged with gender, indicating that gender might not play a significant role in influencing their diversities and relative abundances. However, due to limitations in sample size, further study is needed to confirm these findings. The study also revealed that the most abundant phyla compositions in washing machines were similar, which were Proteobacteria, Bacteroidetes, and Actinobacteria. However, there were differences observed at the species level between males and females, suggesting that the lower the classification level of microbial community structure, the higher the richness of microbial groups. It is worth noting that our study was limited to only 10 samples tested for drug-resistance genes, and the correlation between geographical distribution and ARGs was not clear, which may lead to biased research results. The transmission of ARGs in domestic washing machines occurs through several interconnected processes. The inadequate elimination of microorganisms during washing, particularly at lower temperatures, allows antibiotic-resistant bacteria, potentially carrying ARGs, to persist. These surviving bacteria may form biofilms within the washing machine, providing a protective environment. Contamination from water sources or antibiotic residues in detergents can further support the survival of resistant strains. Cross-contamination during the washing cycle can spread these bacteria, potentially transmitting ARGs to pathogens. Consequently, the household environment may become a reservoir for antibiotic-resistant bacteria, posing a public health risk [5].

## 4. Conclusions

Our study reveals that domestic washing machines in university dormitories harbor a diverse array of microorganisms, including several species with high levels of ARGs. Proteobacteria are the dominant bacteria found in these machines, which exhibited a significant number of ARGs. The majority of the existing ARGs were associated with antibiotic target alteration and efflux, conferring multidrug resistance. More than 130 species were identified, constituting approximately 88% of the total microorganisms present. We found that *Micavibrio aeruginosavorus*, *Aquincola tertiaricarbonis*, and *Mycolicibacterium iranicum* had high levels of ARGs. Mobile genetic elements such as tnpA, intl1 and IS91 were identified as common components facilitating the spread of ARGs.

In conclusion, our findings shed light on the concerning prevalence of antibiotic resistance in domestic environments, with washing machines serving as potential reservoirs for multidrug-resistant bacteria. This underscores the urgent need for enhanced hygiene practices and interventions to prevent the dissemination of antibiotic resistance within university dormitories and beyond. To minimize the risk of spread, it is advisable to follow proper laundry practices, such as using appropriate water temperatures, not overloading the machine, and using detergents as directed. Additionally, regular cleaning and maintenance of washing machines may help prevent the buildup of biofilms and reduce the likelihood of bacterial persistence. However, further research is still needed to enhance our understanding of these processes and refine preventive measures.

## Data Availability

The metagenome sequences were preserved in ENA Sequence Read Archive under accession PRJEB75912.

## Supplementary Materials

The following supporting information can be downloaded at: https://www.mdpi.com/article/10.3390/microorganisms12061112/s1, Figure S1. Diversity and abundance of antibiotic resistance genes (ARGs) in the washing machine. (A) NDMS plot of the ARGs abundance in male and female samples. (B) Comparison of alpha diversity between female and male samples, measured using Chao 1 (left) and Shannon (right) indices. (C) Average abundance of ARGs related to different antibiotics in male and female samples. (D) Average abundance of ARGs related to the microbial resistance mechanisms in male and female samples. Figure S2. Diversity and abundance of mobile genetic elements (MGEs). (A) NDMS plot of the MGE abundance in male and female samples. (B) Comparison of alpha diversity between female and male samples, measured using Chao 1 (left) and Shannon (right) indices. (C) Average abundance of MGEs in male and female samples. Figure S3. Composition and abundance of bacteria at phylum and species level in washing machine. (A) NDMS plot of the bacterial abundance in male and female samples. (B) Comparison of alpha diversity between female and male samples, measured using Chao 1 (left) and Shannon (right) indices. (C) Relative abundance of bacteria at the phylum level.
