# Supplementary material for: Assessing Antibiotic-Resistant Genes in University Dormitory Washing Machines
Source: Microorganisms. 2024 May 30;12(6):1112. doi: 10.3390/microorganisms12061112 (PMC11205806; doi:10.3390/microorganisms12061112)
Supplement: Supplementary file 1 [file microorganisms-12-01112-s001.zip › microorganisms-2998042-supplementary.pdf]

Supplementary Information

Assessing antibiotic-resistant genes in University Dormitory Washing Machines

Wenbo Chen [1, 2, †], Yu Zhang [3, †], Jiandui Mi [1,4, *]

[1] State Key Laboratory for Animal Disease Control and Prevention, College of Veterinary Medicine, Lanzhou University, No. 222 South Tianshui Road, Lanzhou 730000, China.

[2] Division of Bioscience, University College London, London, WC1E 6BT, United Kingdom.

[3] Guangdong Provincial Research Center for Environment Pollution Control and Remediation Materials, College of Life Science and Technology, Jinan University, Guangzhou 510632, China

[4]Gansu Province Research Center for Basic Disciplines of Pathogen Biology, Lanzhou 730000, China.

† These authors contributed equally to this work.

[*] Corresponding authors: mijiandui@163.com

Tel/Fax: 86-020-85280279, 86-13710925973

Address: State Key Laboratory for Animal Disease Control and Prevention, College of Veterinary Medicine, Lanzhou University, No. 222 South Tianshui Road, Lanzhou 730000, Gansu Province, P. R. China

Contents

Figures

Fig. S1. Diversity and abundance of antibiotic resistance genes (ARGs) in the washing machine. (A) NDMS plot of the ARGs abundance in male and female samples. (B) Comparison of alpha diversity between female and male samples, measured using Chao 1 (left) and Shannon (right) indices. (C) Average abundance of ARGs related to different antibiotics in male and female samples. (D) Average abundance of ARGs related to the microbial resistance mechanisms in male and female samples.

Fig. S2. Diversity and abundance of mobile genetic elements (MGEs). (A) NDMS plot of the MGE abundance in male and female samples. (B) Comparison of alpha diversity between female and male samples, measured using Chao 1 (left) and Shannon (right) indices. (C) Average abundance of MGEs in male and female samples.

Fig. S3. Composition and abundance of bacteria at phylum and species level in washing machine. (A) NDMS plot of the bacterial abundance in male and female samples. (B) Comparison of alpha diversity between female and male samples, measured using Chao 1 (left) and Shannon (right) indices. (C) Relative abundance of bacteria at the phylum level.

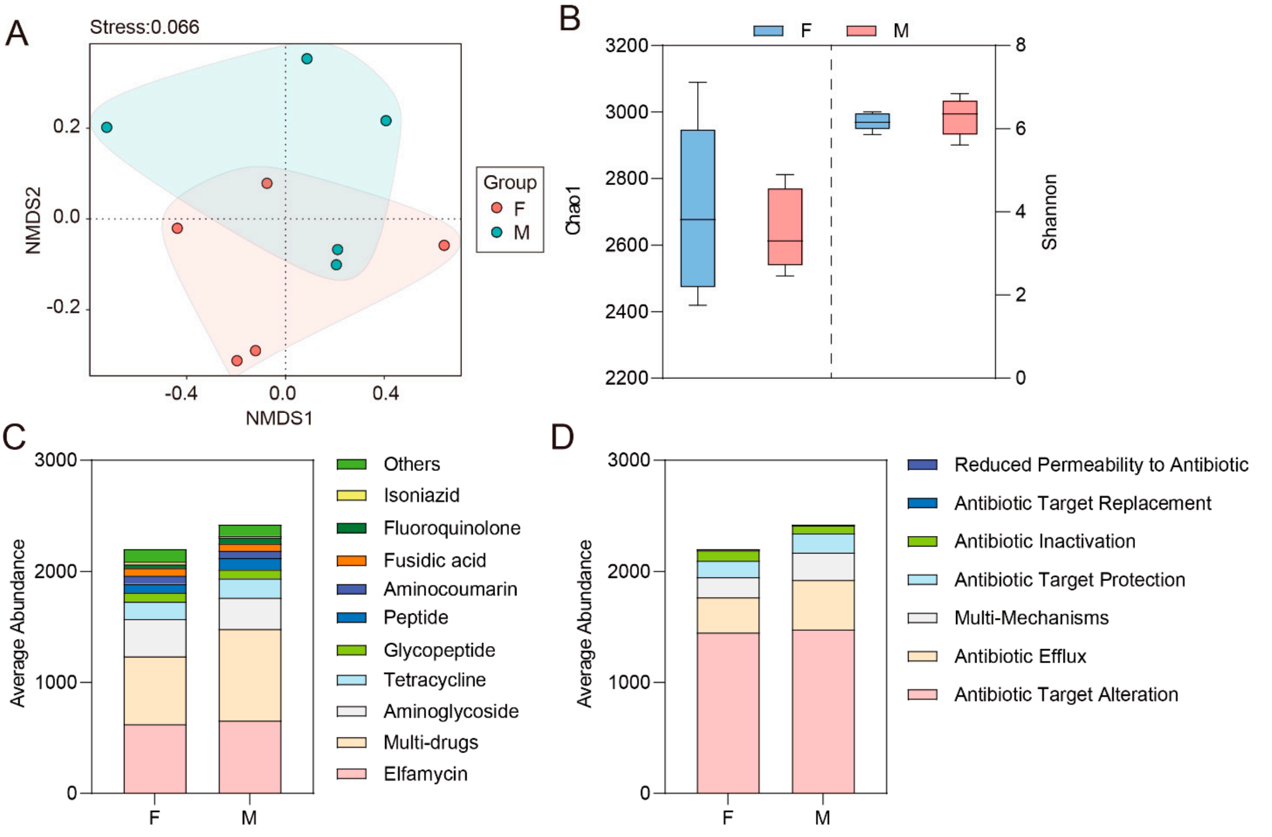

Fig. S1. Diversity and abundance of antibiotic resistance genes (ARGs) in the washing machine. (A) NDMS plot of the ARGs abundance in male and female samples. (B) Comparison of alpha diversity between female and male samples, measured using Chao 1 (left) and Shannon (right) indices. (C) Average abundance of ARGs related to different antibiotics in male and female samples. (D) Average abundance of ARGs related to the microbial resistance mechanisms in male and female samples.

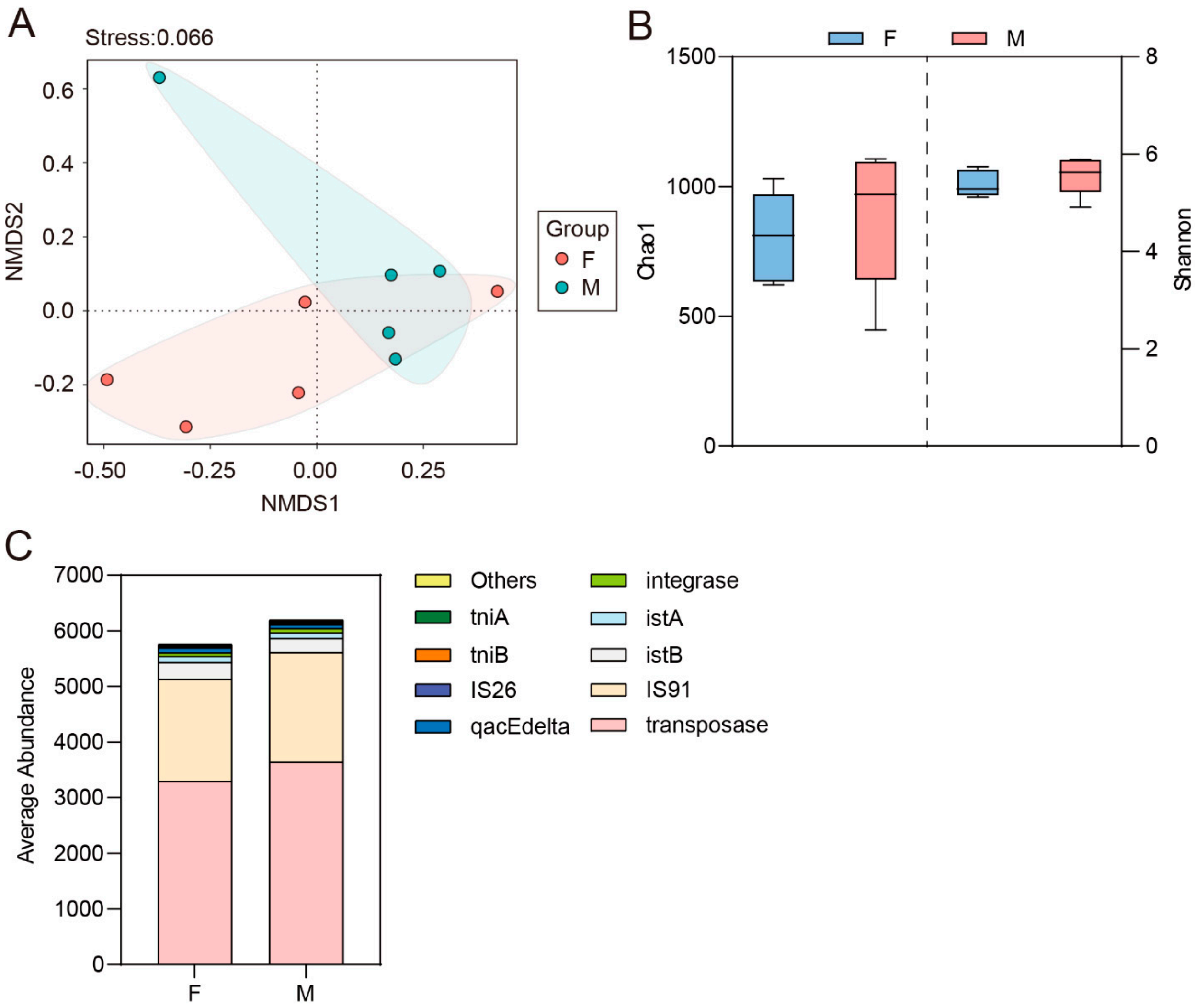

Fig. S2. Diversity and abundance of mobile genetic elements (MGEs). (A) NDMS plot of the MGE abundance in male and female samples. (B) Comparison of alpha diversity between female and male samples, measured using Chao 1 (left) and Shannon (right) indices. (C) Average abundance of MGEs in male and female samples.

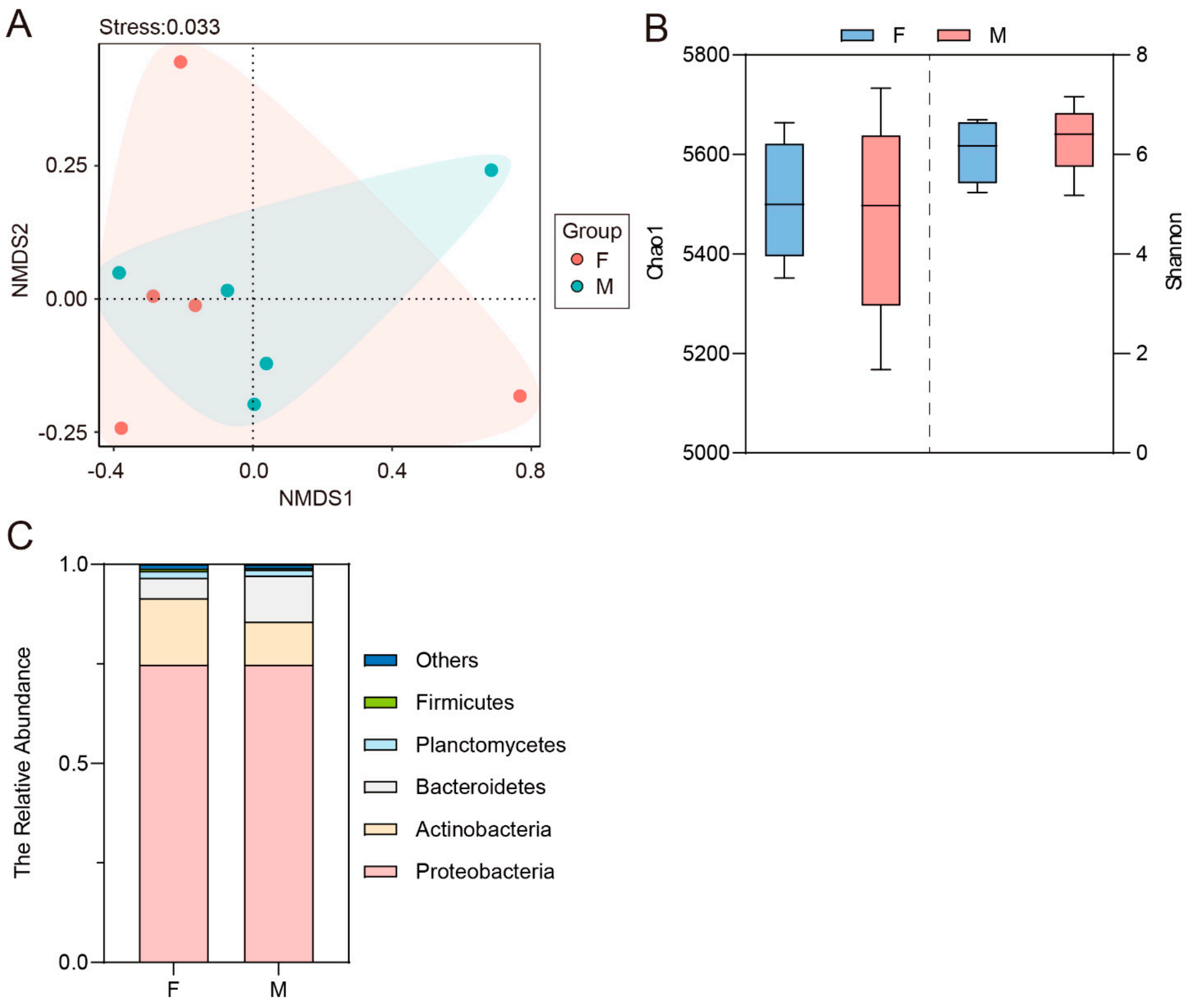

Fig. S3. Composition and abundance of bacteria at phylum and species level in washing machine. (A) NDMS plot of the bacterial abundance in male and female samples. (B) Comparison of alpha diversity between female and male samples, measured using Chao 1 (left) and Shannon (right) indices. (C) Relative abundance of bacteria at the phylum level.
